# A Systematic Review of Sodium-Glucose Cotransporter-2 (SGLT2) Inhibitors in the Management of Heart Failure: A Comprehensive Analysis of Cardiovascular Outcomes, Hospitalizations, and Quality of Life

**DOI:** 10.7759/cureus.86784

**Published:** 2025-06-26

**Authors:** Sumaiya Adrita, Jeffrey Bacsa

**Affiliations:** 1 Department of Emergency, Maidstone and Tunbridge Wells NHS Trust, Kent, GBR

**Keywords:** cardiovascular outcomes, heart failure, hospitalization, quality of life, sodium-glucose cotransporter-2 (sglt2) inhibitors

## Abstract

Sodium-glucose cotransporter-2 (SGLT2) inhibitors have increasingly established themselves as a vital therapeutic option for heart failure across various levels of left ventricular ejection fraction. Recent studies and clinical trials have shown a significant reduction in adverse cardiovascular events and hospitalization rates among patients with heart failure who are treated with SGLT2 inhibitors. However, the long-term implications of these medications for patient survival in heart failure remain uncertain. The primary aim of this review is to assess the effectiveness of SGLT2 inhibitors in managing heart failure, with particular emphasis on cardiovascular outcomes, hospitalization rates, and quality of life.

A systematic search was conducted across multiple electronic databases, including MEDLINE, the Cochrane Library, PubMed, Embase, ClinicalTrials.gov, and CINAHL, to identify high-quality, relevant peer-reviewed studies on SGLT2 inhibitors in heart failure patients. In total, 2,142 records were identified from these databases. After screening, only the records that met the inclusion criteria were retained. Data extraction and analysis were performed by two independent reviewers. Following the screening process, 15 out of the 2,142 studies were included in the final review.

These studies demonstrated the effectiveness of SGLT2 inhibitors in supporting long-term survival by improving cardiovascular outcomes, reducing hospitalization rates, and enhancing quality of life. Three key themes emerged from the research: cardiovascular outcomes (five studies), hospitalization rates (five studies), and quality of life (five studies). Overall, SGLT2 inhibitors are effective in the management of heart failure and contribute positively to the long-term survival of patients. They improve cardiovascular outcomes, decrease hospitalization rates, and enhance the overall quality of life for individuals with heart failure. Nevertheless, additional research with longer follow-up periods and larger sample sizes is needed to establish definitive conclusions regarding the long-term efficacy of SGLT2 inhibitors for survival.

## Introduction and background

Heart failure is a multifaceted clinical condition, marked by the ventricle’s impaired capacity to either pump blood out effectively or fill adequately [1]. In the United States, an estimated 6.7 million adults over the age of 20 are currently affected by heart failure, and this figure is expected to increase to 8.5 million by the year 2030 [2]. Furthermore, the lifetime risk of developing heart failure has increased to 24%, suggesting that one in every four individuals will experience this condition at some point in their lives. 

Despite considerable progress in the management and treatment of heart failure, the condition continues to carry a high burden of mortality and hospital admissions [1]. The unpredictable symptoms of heart failure - including peripheral oedema, fatigue, and breathlessness - often disrupt patients' daily activities and overall quality of life [3]. Additionally, patients frequently become heavily reliant on caregivers and family members, which places an increased burden on both families and the healthcare system. 

As the population continues to age, and the prevalence of comorbidities like diabetes and cardiovascular diseases grows, the prevalence of heart failure is projected to rise accordingly [4]. This underscores the urgent need for effective therapeutic solutions to address this escalating public health challenge.

Sodium-glucose cotransporter-2 (SGLT2) inhibitors represent a class of oral glucose-lowering agents that effectively reduce serum glucose levels and blood pressure by promoting glucosuria [5]. Initially developed as anti-hyperglycaemic medications, SGLT2 inhibitors have demonstrated significant benefits beyond glucose reduction [6-8]. Numerous findings from randomized controlled trials (RCTs) indicate that, in addition to enhancing glucosuria in patients with type 2 diabetes, these inhibitors can markedly reduce hospitalization rates and cardiovascular mortality associated with heart failure [9-13].

The initial cardiovascular outcome trial of empagliflozin in 2013 brought growing recognition to the therapeutic role of SGLT2 inhibitors in heart failure [14]. Further RCTs have reinforced the effectiveness of SGLT2 inhibitors in reducing hospitalizations and improving cardiovascular outcomes associated with heart failure, demonstrating a direct therapeutic effect that is independent of their glucose-lowering properties. This therapeutic impact served as the foundation for several landmark clinical trials in heart failure, irrespective of the patients’ diabetic status. These trials have established the effectiveness of SGLT2 inhibitors in the treatment and management of heart failure in patients [15,16]. A meta-analysis of these studies found a substantial decrease in major adverse cardiovascular events, cardiovascular death, overall mortality, and hospital admissions related to heart failure. The analysis revealed that SGLT2 inhibitors significantly lowered the occurrence of major cardiovascular events, but their effects on cardiovascular mortality showed considerable variation. The findings further indicated that the use of SGLT2 inhibitors lowered the risk of heart failure hospitalization and improved kidney outcomes, with a particularly consistent benefit observed for heart failure risk across numerous RCTs. The notable consistency in these trials underpins the effectiveness of SGLT2 inhibitors in the treatment and management of heart failure, independent of their glucosuric effects [17].

Numerous research studies have reported the positive effects of SGLT2 inhibitors on hospitalization rates and patient-reported outcomes for individuals with heart failure. However, the effects of SGLT2 inhibitors on long-term survival remain unclear [17,18]. Only a limited number of systematic reviews and meta-analyses have evaluated their impact on long-term survival in patients with heart failure and preserved ejection fraction [18,19]. Existing evidence indicates that SGLT2 inhibitors reduce the combined rates of cardiovascular death and hospitalizations, while also enhancing health-related quality of life in this patient population. It is noteworthy that approximately half of all patients with heart failure fall into this category. Currently, there is a gap in the literature regarding the long-term effectiveness of SGLT2 inhibitors in enhancing the quality of life for heart failure patients. This systematic review aims to bridge that gap by evaluating the effectiveness of SGLT2 inhibitors in managing heart failure, specifically concerning cardiovascular outcomes, hospitalization rates, and quality of life. The results of this review will provide a solid foundation for future research focused on the long-term survival of heart failure patients through improved quality of life. Furthermore, it will serve to inform stakeholders and guide clinical decision-making regarding the use of SGLT2 inhibitors in this patient population.

## Review

Methods 

This systematic review was conducted in accordance with the Preferred Reporting Items for Systematic Reviews and Meta-Analyses (PRISMA) guidelines [20]. The PRISMA 2020 Abstract Checklist was utilized to ensure that the review process remained transparent and accurately reflected the authors' work, findings, and their relevance to the primary objective of this systematic review.

Search Strategy

A comprehensive search was conducted across several reputable electronic databases and registries to retrieve high-quality, peer-reviewed research articles pertaining to SGLT2 inhibitors in patients with heart failure. The databases utilized in this search included MEDLINE, the Cochrane Library, PubMed, Embase, ClinicalTrials.gov, and CINAHL. Employing multiple databases is recommended to minimize potential biases in outcomes and findings [21,22]. Keywords were carefully chosen to ensure the retrieval of relevant, high-quality studies. These included: Sodium-Glucose Transporter 2 inhibitors, SGLT2 inhibitors, Canagliflozin, Dapagliflozin, Empagliflozin, Sotagliflozin, Ipragliflozin, Ertugliflozin, Heart Failure, SGLT2 inhibitors AND Heart Failure, Canagliflozin OR Empagliflozin AND Heart Failure, SGLT2 inhibitors AND Cardiovascular Outcome OR Hospitalization Rates OR Quality of Life, as well as the Effectiveness of SGLT2 inhibitors on Quality of Life. Boolean operators “AND” and “OR” were applied to refine the search strategy, enabling a precise and systematic combination of keywords to effectively identify articles relevant to the research objectives and questions. To ensure that no relevant studies or publications were overlooked, manual searches were also conducted in the reference lists of the included research studies.

Inclusion and Exclusion Criteria 

The application of appropriate inclusion and exclusion criteria enhances the credibility, reliability, and validity of the findings in a systematic review [22]. The inclusion criteria for selecting studies in this review consisted of peer-reviewed articles published in English between 2014 and 2024. Additionally, studies were included if they investigated the use of SGLT2 inhibitors in the management of heart failure, regardless of diabetic status. The exclusion criteria encompassed non-peer-reviewed studies, research conducted prior to 2014, studies published in languages other than English, and those focusing on the use of SGLT2 inhibitors in contexts unrelated to heart failure. Furthermore, systematic reviews and meta-analyses were also excluded from consideration in this review.

Data Extraction and Analysis

Research studies identified during the initial search were imported into EndNote X9 (Clarivate Analytics, Philadelphia, PA, USA), and any duplicates were removed. The studies then underwent a thorough and rigorous screening process. This process involved removing duplicates, screening titles and abstracts, and conducting a full-text review. Two independent reviewers carried out these steps, resolving any disagreements through consensus. Data were extracted from texts, figures, tables, and supplementary materials, and were subsequently analysed and synthesized by the reviewers. The content analysis was guided by predefined codes and themes, with a focus on cardiovascular outcomes, hospitalization, and quality of life [23]. Ultimately, three primary themes emerged from the analysis: cardiovascular outcomes, hospitalization rates, and quality of life.

Quality Assessment and Certainty of Evidence 

The research studies included in the systematic review were each assessed individually for quality by two independent reviewers. To evaluate the quality of the 15 studies that met the inclusion criteria, the Cochrane Collaboration’s tool for assessing the risk of bias was employed. This tool encompasses several domains, such as detection bias, selection bias, reporting bias, performance bias, attrition bias, and other forms of bias [24]. The assessment proceeded in three steps: evaluating relevance, identifying concerns regarding the process, and determining the risk of bias [25]. Any disagreements encountered during the quality assessment were resolved by consensus. All 15 studies were classified as either low risk or unclear risk, with none falling into the high-risk category. Additionally, a review of the certainty of evidence for the included studies was performed, utilizing the Five Grades of Recommendation, Assessment, Development, and Evaluation (GRADE) framework [26]. Key considerations for this evaluation included study limitations, publication bias, consistency of effect, imprecision, and indirectness.

Results

Search Results 

**Figure 1:**
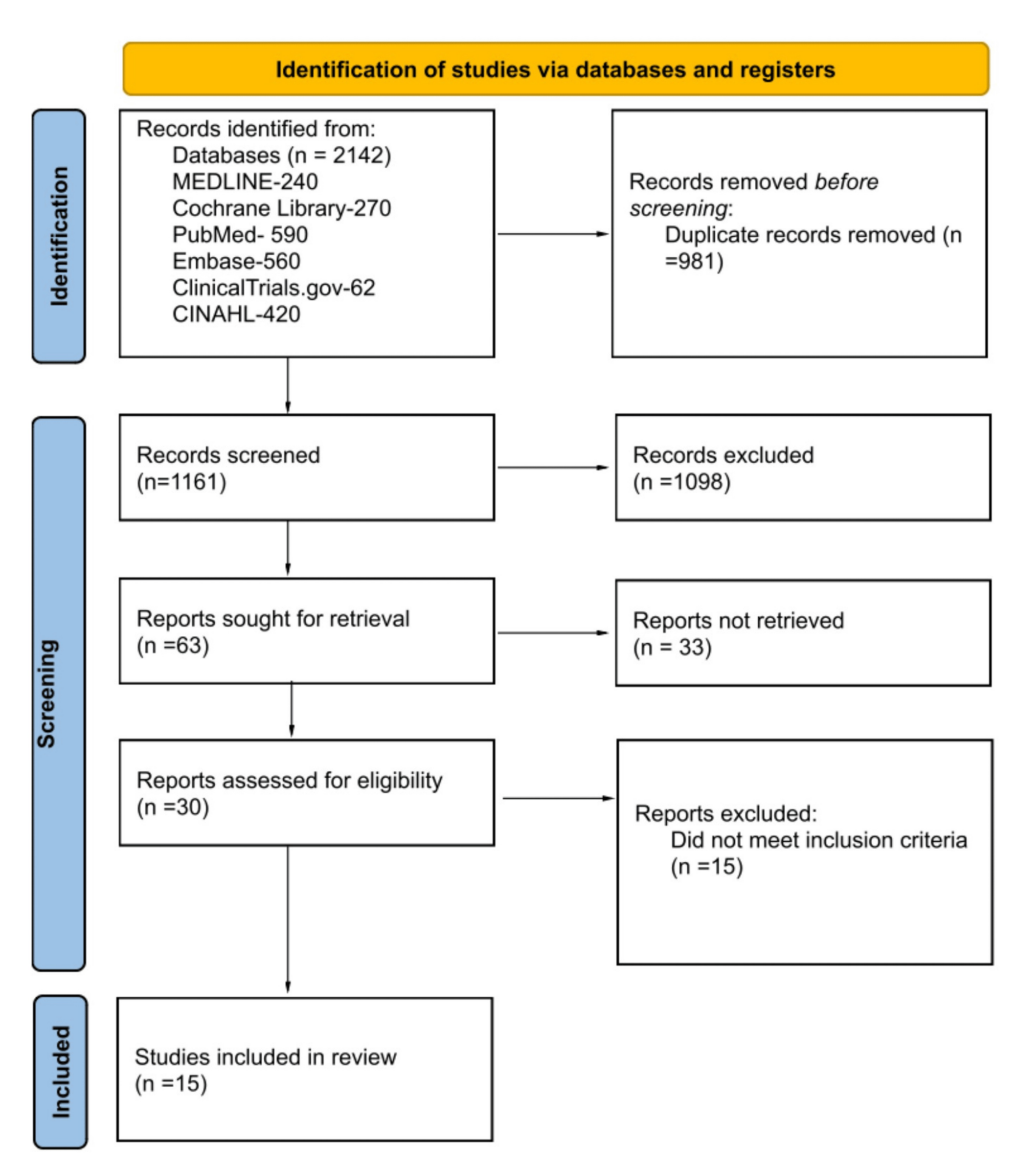
PRISMA Flow Diagram Illustrating the Literature Selection Process in the Systematic Review The PRISMA (Preferred Reporting Items for Systematic Reviews and Meta-Analyses) flow diagram effectively illustrates the literature selection process in systematic reviews. It outlines the quantity of records retrieved from database searches and other sources, the records assessed for eligibility, and the number of studies excluded at each phase. The diagram also highlights the reasons for exclusion and presents the final count of studies included in both qualitative and quantitative syntheses. Each stage is represented by distinct boxes, with arrows indicating the flow of articles from identification to final inclusion. This diagram adheres to the PRISMA 2020 guidelines, ensuring transparency and reproducibility throughout the systematic review process.

Based on the adopted search strategy, a total of 2,142 research studies were identified and obtained. The distribution of records across various databases was as follows: MEDLINE - 240, Cochrane Library - 270, PubMed - 590, Embase - 560, ClinicalTrials.gov - 62, and CINAHL - 420. Of these, 981 records were removed due to duplication. A total of 1,161 records were screened based on their abstracts and titles, resulting in the exclusion of 1,098 records. Consequently, 63 records were sought for retrieval, of which only 30 were successfully obtained. A comprehensive full-text evaluation was conducted on these 30 research studies. Ultimately, 15 studies met the inclusion criteria and were selected for the review process. The PRISMA flow chart diagram detailing this systematic review is presented in Figure 1.

Cardiovascular Outcomes

Five studies have demonstrated the efficacy of SGLT2 inhibitors in the long-term management of heart failure, particularly in improving cardiovascular outcomes. One cohort study investigated the use of SGLT2 inhibitors and their relationship with cardiovascular disease risk in individuals with diabetes. The results indicated that long-term SGLT2 therapy offers significant benefits for patients at higher risk of cardiovascular disease recurrence [27]. This therapy significantly reduced the cardiovascular burden on patients, leading to improved outcomes. In a similar vein, other research studies compared the efficacy of DPP-4 (dipeptidyl peptidase-4) inhibitors and SGLT2 inhibitors regarding the risks of major adverse cardiovascular events [27,28]. The results indicated that the short-term use of SGLT2 inhibitors led to a decreased risk of cardiovascular events [28]. The authors reported enhanced cardiovascular outcomes, including reduced risks of cardiovascular events, mortality, and heart failure.

A multinational randomized trial led by Voors et al. revealed that empagliflozin, an SGLT2 inhibitor, substantially decreased the risk of cardiovascular events, mortality, and heart failure, while also improving the overall quality of life in patients suffering from acute heart failure. These findings corroborated earlier evidence suggesting that SGLT2 inhibitors improve cardiovascular outcomes for individuals with heart failure [29]. Additionally, another study indicated that SGLT2 inhibitors effectively decreased the likelihood of heart failure hospitalization and the incidence of major adverse cardiovascular events. The researchers discussed several potential mechanisms through which SGLT2 inhibitors may lower cardiovascular risk and hospitalization rates for heart failure in both diabetic and non-diabetic patients [30]. 

Moreover, a separate research project aimed to assess the efficacy of SGLT2 inhibitors not only in heart failure but also in the context of type 2 diabetes mellitus and chronic kidney disease. This study concluded that SGLT2 inhibitors significantly reduced incidents of cardiovascular death and heart failure among cohorts with chronic kidney disease, heart failure, and type 2 diabetes mellitus [31]. Collectively, the findings from these five studies highlighted the positive impact of SGLT2 inhibitors on the long-term survival of patients by enhancing cardiovascular outcomes.

Table 1 succinctly summarizes these findings, offering a comprehensive literature matrix of studies assessing the impact of SGLT2 inhibitors on cardiovascular outcomes. It outlines each study’s design, key findings, and specific cardiovascular endpoints, highlighting the consistency and strength of evidence throughout the selected research.

**Table 1:** Literature Matrix Table of Studies on the Impact of SGLT2 Inhibitors on CV Outcomes This table provides a comprehensive summary of studies examining the impact of SGLT2 inhibitors on cardiovascular outcomes. Each row corresponds to a distinct study, with columns outlining essential details, including study design, primary findings, and the specific cardiovascular outcomes evaluated. This table consolidates evidence from multiple sources, providing a clear and concise overview of the association between SGLT2 inhibitor use and cardiovascular health as reported in the literature. It highlights the strength of evidence, overall study quality, and consistency of findings across the included studies. SGLT2, sodium-glucose cotransporter-2; SGLT2i, sodium-glucose cotransporter-2 inhibitors; T2DM, type 2 diabetes mellitus; DPP4i, dipeptidyl peptidase-4 inhibitor; HF, heart failure; CVD, cardiovascular disease; CV, cardiovascular; CKD, chronic kidney disease

Author	Year of Publication	Primary Aim	Research Methodology	Findings
Su et al. [27]	2024	To assess the relative relationship between SGLT2 inhibitors and DPP-4i regarding overall CVD risk in patients with type 2 diabetes in clinical settings.	Retrospective cohort study	The utilization of SGLT2 inhibitors compared to DPP-4 inhibitors was linked to a diminished overall CV load.
Filion et al. [28]	2020	To evaluate the risk of CV events associated with SGLT2 and DPP-4 inhibitors in individuals with type 2 diabetes within a real-world clinical setting.	Multi-database retrospective cohort study	The temporary administration of SGLT2 inhibitors was associated with a diminished risk of CV events in comparison to DPP-4 inhibitors.
Voors et al. [29]	2022	To assess the impact of empagliflozin on three critical objectives of care in patients admitted for acute heart failure.	Randomized controlled trial	The commencement of empagliflozin in patients admitted for acute heart failure is well tolerated and yields substantial therapeutic advantages within 90 days after medication initiation.
Palmiero et al. [30]	2021	To examine the cardioprotective mechanisms of SGLT2i in heart failure patients and their clinical effects on CV events.	Cohort study	The use of SGLT2i seems crucial in clinical practice, particularly in the treatment of HF patients.
Usman et al. [31]	2023	Evaluate the impact of SGLT2 inhibitors on heart failure outcomes and CV mortality among various patient demographics.	Observational study	SGLT2 inhibitors diminish heart failure episodes and CV mortality in populations with HF, T2DM, and CKD, with these effects being constant across patients with different combinations of these conditions.

Hospitalization Rates

Multiple studies indicate that SGLT2 inhibitors play a significant role in the long-term management of heart failure, primarily by reducing hospitalization rates. For instance, a retrospective analysis demonstrated that real-world evidence aligns with clinical trial results, confirming that SGLT2 inhibitor therapy lowers both heart failure incidence and hospital admissions [32]. This reduction can be partly attributed to the ability of SGLT2 inhibitors to alleviate atherosclerotic ischaemic events, which helps to minimize complications such as myocardial infarction in patients with heart failure, ultimately leading to a reduced risk of hospitalization.

Furthermore, research indicates that empagliflozin and dapagliflozin exhibit comparable effects on hospitalization rates and outcomes among patients with type 2 diabetes. However, the effectiveness of these inhibitors may vary, depending on a patient’s history of atherosclerotic cardiovascular disease [33].

These findings corroborate previous studies and underscore the role of SGLT2 inhibitors in the long-term management of heart failure by significantly reducing hospitalization rates [33,34]. By diminishing the risk of heart failure in diabetic patients and lowering the incidence of major cardiovascular events in those with both diabetes and heart failure, SGLT2 inhibitors ultimately decrease unnecessary hospitalizations and enhance the overall quality of life for these individuals. Furthermore, these inhibitors contribute to a reduction in the risk of stroke, myocardial infarction, nonfatal cardiovascular events, and cardiovascular mortality.

A remote, patient-centred, randomized trial demonstrated that canagliflozin, an SGLT2 inhibitor, significantly decreased hospitalization rates among patients with diabetes and heart failure by alleviating symptom burden, thereby enhancing patients' functional status and overall quality of life [35].

In line with these findings, cardiovascular outcome trials have highlighted the efficacy of SGLT2 inhibitors in reducing cardiovascular events, cardiovascular mortality, and hospitalization rates for heart failure [34]. Adding to the evidence of SGLT2 inhibitors' effectiveness in managing heart failure and improving long-term prognosis is their role in modulating mitochondrial function. Specifically, the administration of SGLT2 inhibitors in cardiomyocytes leads to increased plasma levels of ketone bodies, which act as efficient energy sources for the failing heart. These cardioprotective effects are thought to arise from enhanced oxidation of mitochondrial coenzyme Q, and improved free energy availability from cytosolic adenosine triphosphate (ATP) hydrolysis - mechanisms linked to sodium metabolism [36].

As presented in Table 2, the literature matrix highlights several key studies investigating the impact of SGLT2 inhibitors on hospitalization rates. It provides a comprehensive summary of research designs and findings, emphasizing the effectiveness of these agents in decreasing hospitalizations among patients with heart failure and diabetes.

**Table 2:** Literature Matrix Table of Studies on the Impact of SGLT2 Inhibitors on Hospitalization Rates This table summarizes research studies examining the effects of SGLT2 inhibitors on hospitalization rates. Each row corresponds to an individual study, while the columns outline critical study characteristics, including study design and primary outcomes. This organized overview facilitates a comparison of hospitalization outcomes across diverse patient populations and clinical environments. SGLT2, sodium-glucose cotransporter-2; SGLT2i, sodium-glucose cotransporter-2 inhibitors; T2D, type 2 diabetes; HF, heart failure; HFrEF, heart failure with reduced ejection fraction; ASCVD, atherosclerotic cardiovascular disease; EF, ejection fraction; hHF, hospitalization for heart failure; ATP, adenosine triphosphate

Author	Year of Publication	Primary Aim	Research Methodology	Findings
Blanco et al. [32]	2023	To determine if real-world evidence corroborates clinical trial results indicating that SGLT2i decrease hospitalization rates and the incidence of HF among individuals with heart disease and type 2 diabetes.	Retrospective study	Empirical evidence corroborates clinical trial results indicating that SGLT2 inhibitors diminish both the incidence of HF and the rate of hospitalization.
Shao et al. [33]	2021	To investigate the disparities in hospitalization results for hHF following the administration of dapagliflozin or empagliflozin in T2D patients irrespective of a prior history of established ASCVD.	Retrospective multi-institutional cohort study	In T2D patients without ASCVD, dapagliflozin may provide a superior reduction in hHF in comparison to empagliflozin in clinical practice.
Lenahan et al. [34]	2021	To ascertain the function of SGLT2 inhibitors in HFrEF.	Observational study	Medical professionals should prioritize the administration of SGLT2 inhibitors for patients with HF and T2D, as well as specifically utilize dapagliflozin or empagliflozin in patients with HFrEF to enhance cardiovascular outcomes, reduce hospitalizations for HF, and lower all-cause mortality.
Spertus et al. [35]	2022	To ascertain the function of the SGLT2 inhibitor canagliflozin in cardiovascular disease.	Remote, patient-centred randomized trial	Canagliflozin markedly alleviates symptom burden in HF irrespective of EF or diagnosis of diabetes.
Maejima [36]	2020	To examine the existing knowledge regarding the methods by which SGLT2 inhibitors alleviate cardiac dysfunction through mitochondrial protection.	Review of literature	The use of SGLT2 inhibitors increases plasma levels of ketone bodies, which serve as an effective energy source in the malfunctioning heart, by facilitating the oxidation of the mitochondrial coenzyme Q pair and augmenting the free energy of cytosolic ATP hydrolysis.

Quality of Life

Five research studies have demonstrated the effectiveness of SGLT2 inhibitors in managing heart failure by improving patients' quality of life [37-41]. A randomized trial investigating empagliflozin in non-diabetic heart failure patients demonstrated that SGLT2 inhibitors significantly enhanced quality of life by improving left ventricular volumes, mass, systolic function, and functional capacity compared to placebo. Functional capacity improvements were evaluated through validated tools such as cardiopulmonary exercise testing (CPET) and the Kansas City Cardiomyopathy Questionnaire (KCCQ). By positively affecting these cardiac parameters, SGLT2 inhibitors facilitate better symptom management and reduce the recurrence of heart failure, thereby minimizing the risk of long-term complications such as myocardial infarction. These outcomes reinforce the role of SGLT2 inhibitors in the long-term management of heart failure, demonstrating their effectiveness in preventing serious complications like stroke, myocardial infarction, and cardiovascular death [37]. Additionally, a single-centre, real-world observational study indicated that SGLT2 inhibitors effectively reduced all-cause mortality among elderly patients with heart failure with reduced ejection fraction (HFrEF) [38].

A recent study has shown that SGLT2 inhibitors can significantly enhance exercise tolerance in patients with chronic heart failure, which is a crucial factor influencing quality of life and physical functioning. Exercise intolerance is a defining characteristic of heart failure that severely restricts daily activities and independence. SGLT2 inhibitors have been linked to improved exercise capacity, demonstrated through validated assessments such as the KCCQ, six-minute walk test (6MWT), and CPET, resulting in better physical performance and overall patient well-being [39].

Furthermore, a single-centre cohort study revealed that SGLT2 inhibitors positively influenced quality of life by reducing uric acid levels, pulmonary artery pressure, blood pressure, and NT-proBNP (N-terminal pro b-type natriuretic peptide), while increasing body mass index and left ventricular ejection fraction in patients with chronic heart failure. This study further confirmed the efficacy of SGLT2 inhibitors in improving prognosis by decreasing the risk of both primary and secondary endpoints [40].

Moreover, a real-world, multicentre, retrospective cohort study assessed the cardiovascular benefits and safety of SGLT2 inhibitors in patients who have both diabetes and heart failure. The findings indicated that SGLT2 inhibitors markedly improved cardiovascular outcomes in this high-risk population. Consequently, the study recommended incorporating SGLT2 inhibitors into the management strategies for patients with heart failure and diabetes, given their demonstrated ability to enhance quality of life. Moreover, it highlighted the crucial role of SGLT2 inhibitors in reducing adverse events, including strokes, myocardial infarctions, all-cause mortality, acute kidney injury, hypoglycaemia, and urinary tract infections [41].

Table 3 provides a comprehensive overview of studies exploring the impact of SGLT2 inhibitors on cardiovascular outcomes. This table consolidates findings from multiple studies, providing a concise overview of the association between SGLT2 inhibitor use and cardiovascular health as detailed in the reviewed literature.

**Table 3:** Literature Matrix Table of Studies on the Impact of SGLT2 Inhibitors on QoL This literature matrix consolidates studies that explore the effects of SGLT2i on the QoL of patients, especially those suffering from HF and/or T2DM. Each row denotes an individual study, with columns presenting information such as the author and citation, year published, main objective, study design, and principal results. SGLT2, sodium-glucose cotransporter-2; SGLT2i, sodium-glucose cotransporter-2 inhibitors; HF, heart failure; HFrEF, heart failure with reduced ejection fraction; T2DM, type 2 diabetes mellitus; LV, left ventricle/left ventricular; QoL, quality of life; CHF, chronic heart failure; LVEF, left ventricular ejection fraction; NT-proBNP, N-terminal pro B-type natriuretic peptide

Author	Year of Publication	Primary Aim	Research Methodology	Findings
Santos-Gallego et al. [37]	2021	To evaluate the impact of empagliflozin on LV performance, volumes, functional capacity, and QoL in nondiabetic individuals with HFrEF.	Double-blind, placebo-controlled trial	Administration of empagliflozin in nondiabetic patients with HFrEF markedly enhanced LV sizes, LV mass, LV systolic function, functional capacity, and QoL relative to placebo.
Balaguer Germán et al. [38]	2024	To evaluate the impact of SGLT2i on individuals who have HFrEF.	Single-centre, real-world observational study	Administration of SGLT2 inhibitors in elderly patients with HFrEF correlated with a decreased incidence of all-cause mortality.
Voorrips et al. [39]	2022	To ascertain if SGLT2 inhibitors enhance exercise tolerance in progressive HF.	Review of literature	SGLT2 inhibitors enhance exercise tolerance in CHF.
Li et al. [40]	2024	To assess the actual effects of SGLT2i on cardiovascular events, safety, and metabolic profiles in patients with CHF, regardless of the presence of T2DM.	Cohort study	SGLT2i treatment improved LVEF and markedly decreased ambulatory blood pressure, uric acid, fasting blood glucose, pulmonary artery pressure, and NT-proBNP levels in CHF patients.
Kongmalai et al. [41]	2024	To investigate the real-world efficacy and safety of SGLT2 inhibitors on cardiovascular effects in patients with T2DM and HF, as well as to assess the possible relevant adverse event risks.	Retrospective cohort study	SGLT2i inhibitors markedly enhanced cardiovascular outcomes in individuals with T2DM and HF in a real-world context, validating their integration into care strategies for high-risk patients.

Discussion

Based on the findings of this systematic review, SGLT2 inhibitors are effective for the long-term management of heart failure in patients with diabetes and other conditions such as chronic kidney disease. Their clinical benefit is largely attributed to consistent improvements in cardiovascular outcomes, reductions in hospitalization rates, and enhanced patient-reported well-being. Furthermore, short-term administration of SGLT2 inhibitors has demonstrated notable cardiovascular benefits in individuals with heart failure. These medications reduce the cardiovascular burden, lower the risk of major adverse cardiovascular events, and decrease cardiovascular mortality among heart failure patients. Overall, the evidence supports the effectiveness of SGLT2 inhibitors in enhancing cardiovascular outcomes for individuals with heart failure, diabetes, and chronic kidney disease. The results also highlight the effectiveness of SGLT2 inhibitors in reducing hospitalization rates and improving quality of life. Two research studies have indicated that real-world evidence aligns with findings from RCTs, demonstrating that SGLT2 inhibitors lower the incidence of heart failure and related hospitalizations. These agents further contribute to improved quality of life by mitigating the risk of atherosclerotic ischaemic events, including myocardial infarction, stroke, cardiovascular mortality, nonfatal episodes, and chronic kidney disease progression.

SGLT2 inhibitors further diminish hospitalization rates for patients with heart failure and type 2 diabetes mellitus by alleviating symptom burden. One study emphasized that SGLT2 inhibitors enhance quality of life by increasing ketone bodies, which serve as an effective energy source for the failing heart [38-40]. They also provide cardioprotective effects through the modulation of sodium metabolism, improving left ventricular mass, systolic function, and functional capacity [41]. One study offered a complementary perspective, highlighting that SGLT2 inhibitors enhance quality of life by lowering uric acid levels, pulmonary artery pressure, systemic blood pressure, and NT-proBNP concentrations [40].

The results of this systematic review align with recent systematic reviews and meta-analyses that demonstrate the efficacy of SGLT2 inhibitors in patients with heart failure. These findings align with existing evidence demonstrating improved event-free, long-term survival among varied patient populations [42]. They also correspond with research evaluating the safety and effectiveness of SGLT2 inhibitors [43,44]. Although the two systematic reviews targeted heart failure with mildly reduced or preserved ejection fraction, their findings can be generalized and support the conclusions of this review [43,44]. These reviews offer a crucial perspective on the long-term safety of SGLT2 inhibitors, highlighting their favourable risk-benefit profile.

In addition, the conclusions drawn in this review are in strong agreement with those of the systematic review conducted by Tsampasian et al. [45]. Although there were variations in the electronic databases used for study selection, both reviews yielded comparable evidence indicating that SGLT2 inhibitors significantly improve cardiovascular outcomes and reduce mortality rates.

This review also supports the growing body of evidence suggesting that SGLT2 inhibitors offer a promising therapeutic option for patients with heart failure with preserved ejection fraction, demonstrating a favourable safety profile. Their favourable safety profile, combined with consistent improvements in quality of life and reductions in cardiovascular mortality and heart failure-related hospitalizations, echoes results from other recent systematic evaluations and meta-analyses [45].

Limitations

This systematic review has several limitations, including the inclusion of varied study designs, such as cohort, retrospective, and observational studies, alongside RCTs. It is worth noting that numerous recent systematic reviews prioritize exclusively RCTs to maintain a superior level of evidence. Furthermore, the review utilized two independent reviewers for data compilation, extraction, and thematic analysis. The involvement of different reviewers can introduce varying perspectives and interpretations of the results and findings.

Another potential limitation is the small sample size of the primary sources that contributed data for this review. Only 15 research studies met the inclusion criteria and were ultimately included. Although the findings may be generalizable to other populations, increasing the sample size would offer more definitive evidence to either validate or challenge the results of this review.

## Conclusions

The primary objective of this systematic review was to assess the effectiveness of SGLT2 inhibitors in the management of heart failure, with a particular emphasis on cardiovascular outcomes, hospitalization rates, and quality of life. The evidence gathered indicates that SGLT2 inhibitors are effective in managing heart failure and improving the long-term survival of patients by enhancing cardiovascular outcomes, reducing hospitalization rates, and promoting overall quality of life. Research has shown that the benefits of SGLT2 inhibitors in managing heart failure are evident across various patient populations and are independent of their glucosuric effects in the body. Nevertheless, further studies with longer follow-up periods and larger populations are needed to confirm the sustained benefits of SGLT2 inhibitors on cardiovascular outcomes, hospital admissions, and quality of life in heart failure patients. Incorporating meta-analyses of RCTs in future research will be essential to validate or challenge the conclusions drawn from this systematic review.
